# Acute Gout.—II

**Published:** 1908-02-15

**Authors:** 


### Clinical Points.

### ACUTE GOUT.—II.

The following is a brief discussion upon some of the more salient points in a case of acute gout with gastric and bronchial symptoms, described on page 495.

The first point deserving notice is the accumulation of urate of soda as "chalky" deposits in certain places. These accumulations, in the case refererd to, took place in the ears, and about the small joints of the upper extremities; and this is almost always the case. Although the lower extremities do not wholly escape, still the great accumulation is generally in the upper ones, and the quantity found in the lower ones is usually small.

The deposit takes place in greatest abundance in the subcutaneous tissue. In some instances it forms a hard, dense mass, intersected by the bands of fibres of the areolar tissue; but in others these bands of fibres seem to be absorbed, and a cavity is formed, filled wholly with this urate of soda, which may be easily dislodged when the skin is freely opened. At the same time it is, of course, often found in joints, seeming to smear the surfaces of the articular cartilages and making them rough, so that they grate against each other. It will also make its way into the interstices of the fibres of ligaments and tendons, and stiffen them. Another very common place for these deposits is the ear, beneath the skin covering the cartilages; and it sometimes occurs over the cartilages of the alæ nasi. Certain bursæ may also be the site of the deposit, the bursa most commonly affected being that over the olecranon process of the ulna bone. Sometimes the water with which the deposit is mingled, and which gives it its soft pasty character, becomes absorbed, and a dry "chalk-stone" is formed, often as big as a marble, which will leave a white mark on a blackboard.

Another curious point to bear in mind about these deposits is that, when they occur at all, they nearly always do so early in the disease. The man whose case has been given was under forty when they came on, and he had not been more than five years the subject of gouty attacks before these accumulations had taken place to a considerable extent. There are many instances of patients having had paroxysm after paroxysm of gout for a long series of years without any marked accumulation at all; joints may be damaged, cartilages altered, ligaments and tendons stiffened; but the areolar tissue will be free from any collections of urate of soda. On the other hand, in the type of case we have described, each fresh paroxysm is accompanied by a new deposit, or a greater or less addition to those already existing. Moreover, even though the fresh attacks occur in the legs, the additions of urate of soda are still mainly in the hands.

The patient had in some way acquired a secondary infection in the last attack described. Uratic deposits may exist for years and years without suppurating. Each is, however, a *locus resistentiæ minoris* in which, if a few staphylococci acquire entrance, suppuration may readily ensue, as in his case.

The man afforded an excellent opportunity of studying the clinical phenomena of gout when it attacks the stomach or bronchial tubes. The symptoms which indicated that the stomach was attacked were the severe and incessant vomiting, the pain in the region of the stomach, which at times was agonising, and the flatulent distension of the organ. These symptoms recurred in consecutive attacks. When the stomach is affected with gout its mucous membrane appears to become highly irritable, and for some reason gas is formed with great rapidity, distending the viscus. It is this great inflation of the stomach which probably, in part at least, causes the violent pains which patients suffer during the attack; for when they are able to expel the wind in considerable quantity the pain becomes much less, or entirely disappears. The ability to get the wind up is much impaired, however; the distension of the organ seems to interfere with the muscular power of its walls, and when vomiting or belching occurs it is largely brought about by powerful contractions of the abdominal muscles. Frequent repetition of this process in turn leaves behind it a painfulness of the abdominal parietes.

In the case described, the stomach was attacked simultaneously with the other parts. The selfsame symptoms, as regards the stomach, may be seen sometimes after the suppression of external gout by colchicum, or precede the development of it in the foot or some other part. In the latter cases the diagnosis of the nature of the abdominal symptoms may be exceedingly difficult until the joint troubles declare themselves. Exactly what happens in the tissues of the stomach during such an attack is quite unknown. Urate of soda deposits do not occur there. Post-mortem examinations are exceedingly rare, because the patients very seldom die when an attack of the kind is in progress.

The patient also showed how the bronchial tubes may become affected in acute gout. While the hands and forearms and other external parts were still suffering the breathing became difficult, wheezy, and more rapid, rhonchi and prolonged expiration were heard all over the chest, and a troublesome cough came on, accompanied with frothy expectoration. Similar bronchitic signs were associated with each of several different attacks of gout, so that the bronchitis can scarcely be regarded as accidental. The very same symptoms will often be found to precede the external development of gout, or to follow its recession from some external part, just as in the stomach affection.

### Treatment.

In regard to the treatment of the case, the main point of practical interest is in connection with the alleviation of the gastric symptoms which were so extremely severe. The remedies which undoubtedly did most good were opium, the application of free couter-irritation over the epigastric region by mustard and by turpentine, and the exhibition of carbonate of ammonia in effervescence, taking care to allow three or four grains of the ammonium salt to remain in excess at each dose. The opium was given at night in one dose, rather than in repeated doses during the day, in order to obtain sleep at the natural time. For two nights it was combined with two grains of the acetic extract of colchicum; but this was very soon given up, as it seemed to depress the patient. The effervescing ammonia was exhibited frequently during the day, as often as every two or three hours; and when the trouble was at its height fifteen minims of chloric ether—a very grateful and valuable stimulant—was added to each dose.

At the some time it was found necessary to give brandy in small and frequent doses, and nothing seemed to remain upon the stomach better than this. At first the patient took two drachms every hour; but it was afterwards found necessary to increase it to half an ounce. The most obvious proof of the need for this kind of treatment was afforded by diminishing the allowance of brandy one day, when the man seemed a little improved in strength, from half an ounce per hour down to two drachms. After he had been twenty-four hours on this diminished allowance, he became very much reduced in strength, his tongue parched and dry, and his pulse quickened; but these symptoms soon disappeared on putting him on the larger allowance again. This time two drachms were given every half-hour instead of half an ounce every hour—a mode of giving stimulants in debilitating maladies which is attended with the happiest results. After three days of this treatment the irritability and pain of the stomach had completely yielded; but on account of his general condition it was thought advisable to continue it for a fortnight, especially when the bronchitis came on. This latter affection was combated by free counter-irritation, with turpentine stupes to the chest, both in front and behind, and afterwards by a blister to the sternum.

It may seem somewhat anomalous that the treatment of a gouty state of stomach should consist in the administration of stimulants like ammonia and brandy, the intemperate use of which, according to popular belief, tends rather to generate the gouty condition. It might be argued that, if there be any state of inflammation of the mucous membrane of the stomach at all like the external inflammation witnessed about the joints of the upper extremities, surely the application of such hot things as ammonia and brandy must do harm. So it would seem reasonable enough, *à priori*, to assume; but, unfortunately, in the practice of medicine, *à priori* reasoning is often of less value than actual experience. Experience returns an unequivocal verdict in favour of this plan of treatment for the acute gastric disorder that is sometimes associated with gout.

Nor is this without analogy; for in many forms of conjunctivitis—and among them in that which is associated with a gouty state—great benefit will be obtained from the application of tincture of opium and of a solution of nitrate of silver to the eye.

